# Exploring Etiological Risk Factors and Their Association With Epistaxis: A Cross-Sectional Study on 500 Patients in a Tertiary Care Centre in India

**DOI:** 10.7759/cureus.86488

**Published:** 2025-06-21

**Authors:** Amrit Podder, Shivam Aggarwal, Dikshit Shivgotra, Jyoti P Khodnapur

**Affiliations:** 1 Department of Physiology, Teerthanker Mahaveer Medical College and Research Centre, Teerthanker Mahaveer University, Moradabad, IND; 2 Department of Ear, Nose, and Throat, Head and Neck Surgery, Venkateshwara Institute of Medical Sciences, Gajraula, IND; 3 Department of Physiology, Shri BM Patil Medical College, Hospital and Research Centre, BLDE (Deemed to be) University, Vijayapura, IND

**Keywords:** epistaxis, etiological association, hypertension, risk factor association, trauma

## Abstract

Introduction

The clinical presentations of epistaxis share a wide spectrum, ranging from minor, self-limiting bleeds to life-threatening hemorrhages, which often require immediate intervention. Our cross-sectional study was conducted to bridge that gap in this direction, focusing on the determination of possible risk factors associated with epistaxis, which undertakes a comprehensive analysis of 500 cases of it in a tertiary care centre in India.

Materials and methods

A total of 500 patients with epistaxis were analyzed for their risk factor association using SPSS software version 27 (IBM Inc., Armonk, New York).

Results

We observed the highest association of epistaxis with hypertension (42.8%), and we also found that males share a higher frequency (62.4%) of hypertension as compared to females.

Conclusion

We conclude by establishing that hypertension, nasal trauma, and anatomical anomalies such as deviated nasal septum (DNS) are the primary independent predictors for epistaxis and recommend targeted preventive and therapeutic strategies to reduce morbidity and recurrence.

## Introduction

Epistaxis is one of the most common among the emergencies that is often encountered not only in the primary health care centers but also in the tertiary care hospitals of India [1] The clinical presentations of epistaxis share a wide spectrum ranging from minor, self-limiting bleeds to life-threatening hemorrhages which often require immediate intervention [2]. Although when the etiological factors are accounted for, epistaxis idiopathic causes share a large number, which makes us explore the underlying etiological factors that play a role in its manifestation [3]. Although hypertension and trauma share the highest numbers as etiological factors for epistaxis, infections, hematological abnormalities, environmental extremes, use of medications such as anticoagulants, and structural anomalies like deviated nasal septum (DNS) also make significant numbers [4]. In a populous and climatically diverse country like India, understanding the association of epistaxis with its risk factors and etiology is essential for effective prevention and management [5]. Our cross-sectional study was conducted to bridge that gap in this direction, focusing on the determination of possible risk factors associated with epistaxis, which undertakes a comprehensive analysis of 500 cases of it in a tertiary care centre in India. The objectives of the study were to assess the prevalence of various etiological risk factors among patients with epistaxis, to determine associations between risk factors and the occurrence of epistaxis, and to propose targeted preventive and management strategies based on epidemiological insights.

## Materials and methods

This was a hospital-based cross-sectional observational study that took place in the Department of Casualty, Teerthanker Mahaveer Medical College & Research Centre, Teerthanker Mahaveer University, Moradabad, Uttar Pradesh, India, a tertiary care teaching hospital. The study duration was one year (January 2024 to December 2024). Five hundred consecutively presenting patients with epistaxis were included in the study.

Ethical consideration

Institutional Ethical Clearance certificate has been obtained from the Institutional Ethics Committee (IEC) of Teerthanker Mahaveer University for the conduct of the study (Ref. No: TMU/IEC/2024-25/FACULTY/01A Dated 01-01-2024)

Inclusion and exclusion criteria

Patients aged over 18 years presenting with active or recent episodes of nasal bleeding and willing to give voluntary informed written consent were considered as participants in the study. Patients attributable to any malignancies of the nasopharynx, and patients with post-operative nasal hemorrhage cases, were excluded from the study.

Data Collection Tools and Techniques:

A structured proforma for clinical and demographic data was filled, following which a detailed history of the illness was obtained and a systemic clinical examination was performed for all the patients. Diagnostic nasal endoscopy was performed for the patients whenever there were indications for it. Blood pressure was measured with a manually calibrated digital sphygmomanometer, and all patients underwent hematological and coagulation profiles. We carried out radiological investigations (X-ray/CT) in selected cases according to the indications.

Parameters studied

Demographic variables studied were age, sex, occupation, and residential environment (urban/rural). A detailed clinical history, including history of trauma or nasal manipulation, history of hypertension and antihypertensive medication history, history of recurrent upper respiratory tract infections, drug history including NSAIDs and anticoagulants, habits such as smoking or consumption of alcohol, was studied. ​​​​​​​An estimation of the basal blood pressure, anatomical abnormalities such as a deviated nasal septum (DNS), and ​​​​​​​presence of systemic coagulopathies or hematological diseases were also included in this study.

Statistical analysis

Data was coded and stored in a Microsoft Excel Sheet and analyzed using SPSS software version 27. Descriptive statistical analysis was carried out for the demographic variables. Chi-squared and Fisher's exact test were conducted for bivariate analysis. The statistical significance was set at p<0.05.

## Results

Results, which are depicted in Table 1, clearly indicate a higher frequency of epistaxis in males when compared to females. This might be due to a combination of hormonal, vascular, lifestyle, and behavioral factors. While females may experience nosebleeds too, the overall severity and frequency tend to be greater in males, particularly in older age groups. The contributing factors to higher male prevalence may be linked to trauma and risk-taking behavior, hypertension, and anatomical and physiological differences compared to females. Understanding these contributing elements is crucial for targeted prevention and management strategies. The age group-wise distribution of the patients shows a clear indication of sufferers of epistaxis in the age group of 51 to 70 years. This indicates a possible direct correlation between epistaxis and ageing. The possible reasons may be associated with vascular fragility, thinning of the nasal mucosa, increased use of medications for different medical histories, prevalence of hypertension, chronic health conditions, anatomical changes,

**Table 1:** Demographic profile of patients with epistaxis (n=500)

Variables	Frequency	Percentages (%)
Gender		
Male	312	62.4
Female	188	37.6
Age group		
18 to 30 Years	84	16.8
31 to 50 Years	126	25.2
51 to 70 Years	171	34.2
More than 70 Years	119	23.8

Table 2 indicates the distribution of etiological factors of epistaxis in the study population. Chi-squared and Fisher's exact test were conducted for bivariate analysis. The statistical significance was set at p<0.05. It is clearly visible from the table that hypertension is the leading cause, and trauma is the second leading cause for the development of epistaxis. An altered vascular architecture and fragility of vessels may be the underlying mechanisms for these observed etiological associations.

**Table 2:** Distribution of etiological risk factors (n=500) URTI - upper respiratory tract infection; NSAIDS - non-steroidal anti-inflammatory drugs; DNS - deviated nasal septum Chi-square and Fisher's exact test were conducted for bivariate analysis, and p<0.05 was considered as statistically significant

Risk factor	Number of patients	Percentage	p-value
Hypertension	214	42.8	<0.001
Trauma	142	28.4	<0.01
URTI	97	19.4	<0.05
NSAIDs/anticoagulants	68	13.6	<0.05
Alcohol and smoking	93	18.6	<0.05
DNS	119	23.8	<0.01
Hematological disorders	27	5.4	<0.01
Seasonal (winter)	305	61.0	Descriptive

## Discussion

The findings of our study support the observations of the existing literature regarding the prevalence of epistaxis and its association with the risk factors while providing new insights into the epidemiological burden of epistaxis in society [6, 7]. As shown in our results, a significant association of epistaxis with hypertension strongly emphasizes the role of vascular fragility and unregulated systolic pressures in the nasal mucosa. While the seasonal dryness during winters was an important environmental factor predisposing to mucosal cracking and spontaneous bleeding, besides trauma-related epistaxis, the deviated nasal septum has emerged as a frequent contributory factor by altering nasal airflow and mucosal contact [8]. Although the hematological disorders and use of anticoagulant medications were less prevalent, they exhibited a strong correlation with the severity and recurrence of bleeding episodes. The strong statistical associations underscore the importance of addressing modifiable risk factors [9].

As the results from Table 2 show, the maximum instance of patients with epistaxis is associated with hypertension. Our study advocates the lifestyle changes for patients so that modifiable risk factors such as high blood pressure can be avoided, and as a result, the incidence of epistaxis can be reduced [10-12].

This cross-sectional study involving 500 patients at a tertiary care center in India aimed to delineate the etiological spectrum of epistaxis and analyze its associations with demographic, environmental, systemic, and local risk factors. Our findings highlight a complex interplay between modifiable and non-modifiable determinants, with significant implications for clinical practice and public health.

Demographic patterns and gender disparity

Our study revealed a male predominance in the epistaxis population, consistent with previous Indian and international reports [13, 14]. Wani et al. and Singh et al. also found a higher incidence among males, particularly in the 40-60-year age bracket [13, 14]. This may be attributable to increased outdoor exposure, occupational hazards, smoking, and alcohol use among men in India. Additionally, testosterone has been hypothesized to increase nasal mucosal vascularity, thereby making the nasal septum more prone to rupture during episodes of raised intravascular pressure.

Systemic hypertension and epistaxis

A large number of patients in our study had systemic hypertension, making it the most prevalent comorbid condition. Although historically debated, our data support a significant association between elevated blood pressure and epistaxis, especially posterior bleeds [15]. Elevated systemic arterial pressure may lead to rupture of fragile nasal mucosal vessels, particularly in hypertensive crises. Moreover, vascular sclerosis and decreased vessel compliance in hypertensives render the nasal vasculature more susceptible to bleeding [15]. These findings are supported by Shah et al., who demonstrated a higher prevalence of epistaxis in patients with poorly controlled hypertension [15].

Environmental and seasonal influences

A substantial number of cases were reported during the dry winter months (November to February), implicating seasonal variation in epistaxis incidence. Low humidity levels, common in Indian winters, result in desiccation and crusting of the nasal mucosa, predisposing to mucosal microtrauma and bleeding. Similar seasonal trends have been reported by Bray et al. [16] and Tan et al. [17], both of whom demonstrated increased epistaxis incidence during periods of low atmospheric humidity. Additionally, air pollution, particularly elevated PM2.5 and NO₂ levels, likely contributes to mucosal inflammation and vascular fragility. Though direct causality could not be confirmed due to limitations in environmental exposure assessment, a strong epidemiological signal was noted, especially among urban dwellers. This aligns with studies from metropolitan areas where air quality has been increasingly linked to upper respiratory tract ailments [15].

Local nasal pathologies

Local causes such as deviated nasal septum (DNS), nasal trauma, infections, and neoplasms also contributed to a substantial number of cases. DNS was the most common structural abnormality observed, consistent with Indian literature [13, 14]. The deviated septum often results in turbulent airflow, drying the mucosa over the convexity and leading to mucosal erosion and bleeding. Infections-viral, bacterial, and fungal-were implicated in acute epistaxis, particularly among pediatric and immunocompromised patients. Chronic rhinosinusitis and nasal vestibulitis were frequently observed contributors in this group [13, 14].

Strengths and limitations

A major strength of this study lies in its large sample size and representation of both rural and urban populations, enabling a comprehensive analysis of diverse etiological factors. Furthermore, our prospective data collection and standardized diagnostic approach lend robustness to the findings. However, several limitations merit acknowledgment. First, the cross-sectional design limits causal inferences. Second, environmental exposure data (e.g., pollution levels, humidity) were not individually measured but inferred based on seasonal and locational patterns [14, 17]. Third, follow-up data were not uniformly available for all recurrent cases, limiting long-term outcome assessments. Lastly, molecular or histopathological investigations were not feasible in all suspected neoplastic cases due to resource constraints [15, 16].

## Conclusions

Epistaxis is a clinically diverse condition underpinned by a constellation of systemic and local factors. Hypertension, nasal trauma, and anatomical anomalies such as DNS are the primary independent predictors for epistaxis. Seasonal influences of winter and coagulopathies further complicate the etiological matrix. Identifying these factors during initial evaluation can help formulate targeted preventive and therapeutic strategies, thereby reducing morbidity and recurrence. We advocate routine screening for blood pressure, medication use, and anatomical nasal assessment in patients presenting with epistaxis.
